# Polymicrobial Interactions in the Cystic Fibrosis Airway Microbiome Impact the Antimicrobial Susceptibility of *Pseudomonas aeruginosa*

**DOI:** 10.3390/antibiotics10070827

**Published:** 2021-07-07

**Authors:** Emma Reece, Pedro H. de Almeida Bettio, Julie Renwick

**Affiliations:** Department of Clinical Microbiology, Trinity College Dublin, D02 PN40 Dublin, Ireland; ereece@tcd.ie (E.R.); bettiop@tcd.ie (P.H.d.A.B.)

**Keywords:** cystic fibrosis, *Pseudomonas aeruginosa*, polymicrobial interactions, microbiome, antibiotic resistance

## Abstract

*Pseudomonas aeruginosa* is one of the most dominant pathogens in cystic fibrosis (CF) airway disease and contributes to significant inflammation, airway damage, and poorer disease outcomes. The CF airway is now known to be host to a complex community of microorganisms, and polymicrobial interactions have been shown to play an important role in shaping *P. aeruginosa* pathogenicity and resistance. *P. aeruginosa* can cause chronic infections that once established are almost impossible to eradicate with antibiotics. CF patients that develop chronic *P. aeruginosa* infection have poorer lung function, higher morbidity, and a reduced life expectancy. *P. aeruginosa* adapts to the CF airway and quickly develops resistance to several antibiotics. A perplexing phenomenon is the disparity between in vitro antimicrobial sensitivity testing and clinical response. Considering the CF airway is host to a diverse community of microorganisms or ‘microbiome’ and that these microorganisms are known to interact, the antimicrobial resistance and progression of *P. aeruginosa* infection is likely influenced by these microbial relationships. This review combines the literature to date on interactions between *P. aeruginosa* and other airway microorganisms and the influence of these interactions on *P. aeruginosa* tolerance to antimicrobials.

## 1. Introduction

It is estimated that approximately >70,000 people are living with cystic fibrosis (CF) worldwide [1]. Despite being a multi-organ disease affecting the pancreas, sweat glands and intestines, airway infections and the associated inflammation are the largest contributors to morbidity and mortality in CF [2]. Even with modern medical advances, the median life expectancy for people with CF is approximately 40 years [3]. CF is caused by mutations in the CF transmembrane conductance regulator gene. Lack of functional CFTR protein results in defective secretion of Cl^−^ with enhanced Na^+^ absorption and mucus secretion [4]. The airway surface fluid becomes viscous, which leads to impaired mucociliary clearance, creating an ideal environment for colonisation of microorganisms. CF airway disease is characterized by a continuous cycle of persistent infection and inflammation culminating in reduced lung function and eventually respiratory failure.

The most commonly cultured bacteria from CF airway samples during periods of exacerbation are *Pseudomonas aeruginosa*, *Staphylococcus aureus*, *Haemophilus influenzae*, *Stenotrophomonas maltophilia*, non-tuberculous mycobacteria, and *Burkholderia* species [5,6]. *P. aeruginosa* is an important opportunistic pathogen that causes acute infections in immunocompromised patients. *P. aeruginosa* has robust virulence systems and is capable of forming multidrug-resistant biofilms which allow for persistent colonisation of the CF airways; thus, *P. aeruginosa* is the most common cause of exacerbation and mortality in people with CF [7,8]. Early intervention is paramount as *P. aeruginosa* is very difficult to eradicate once it has established chronic colonisation of the airways. *P. aeruginosa* infection is linked to decreased life expectancy of 30 years, compared with 40 years in non-colonised patients, and these patients experience a more rapid decline in pulmonary function with more frequent hospitalisations [9,10]. Chronic *P. aeruginosa* infection is associated with antibiotic resistance [11,12], poor response to antibiotic therapy [12], rapid decline in lung function, and poor clinical outcomes [13,14,15,16].

With the advent of next-generation sequencing (NGS) and the discovery of a diverse community of microorganisms in the CF airway, it is now known that *P. aeruginosa* does not colonise the airways alone. Inter-species, cross-genera, and cross-Kingdom interactions have been shown to influence pathogen virulence [17,18,19], reduce susceptibility to antibiotics [20,21], and impact patient health [17,22,23]. The virulence of *P. aeruginosa* is influenced by its co-colonisers in the CF lung microbiome [24,25,26]. Several studies have now shown that *P. aeruginosa* responds differently to antibiotics when cultured alone and in co-culture with other microorganisms [20,21,27,28]. Furthermore, clinical trials have shown a lack of association between clinical response to antibiotic therapy and in vitro susceptibility of the cultured bacteria [29,30]. Changes in virulence and antimicrobial susceptibility in polymicrobial communities have the potential to drive poorer clinical outcomes in CF.

In this review, we focus on the impact of polymicrobial interactions on *P. aeruginosa* resistance/tolerance to antibiotics and the potential implications this has on therapeutic approaches in CF.

## 2. *P. aeruginosa* Infection in CF

*P. aeruginosa* infection is known to transition from sporadic colonisation to an intermittent infection before becoming established, and approximately 80% of CF patients are chronically colonised by *P. aeruginosa* by the age of 20 [31,32]. Development of chronic *P. aeruginosa* colonisation is a significant disease milestone in CF and has been observed to mark the beginning of significant lung function decline [33]. Chronic *P. aeruginosa* colonisation is considered a major limiting factor in patient survival, with estimates of 10 years reduced life expectancy in chronically colonised patients [9,10,31]. Early diagnosis and eradication are extremely important in delaying chronic infection. *P. aeruginosa* has several virulence factors that contribute to colonisation and airway inflammation within the CF lung, including Type III secretion system (T3SS) [34], lipopolysaccharide (LPS) [35], exopolysaccharide (EPS) [36], proteases [37,38], pyocyanin [39], and siderophore production [40]. Some of these virulence factors directly damage host tissue [35] and redirect immune cell functions [41] and can specifically target neutrophils [37,42]. Microorganisms exhibit social behaviours and live-in communities termed biofilms, which are embedded in EPS which protects them from the surrounding environment. The majority of *P. aeruginosa* EPS are Psl, Pel, and alginate; these play distinct roles in attachment and biofilm formation [43]. *P. aeruginosa* planktonic cells attach to the surface initially through weak and reversible adhesion, followed by formation of microcolonies [44]. The cells then embed themselves in a matrix of EPS which comprises proteins, lipids, and nucleic acids to facilitate maturation [45]. The EPS in biofilms affects the structure, including its density and stability. Finally, there is the dispersal stage where cells from the microcolonies leave the biofilm and colonise a new surface. *P. aeruginosa* can form biofilms that are associated with multidrug resistance (MDR) [46]. *P. aeruginosa* biofilms have been shown to be up to 1000 times more resistant to antimicrobials than planktonic cells [47,48]. There are numerous mechanisms involved in biofilm resistance, including adaptive stress responses, reduced penetration, and increased horizontal transfer of antibiotic resistance genes (ARGs) [27,49,50]. Biofilm antimicrobial tolerance results from restricted penetration of antimicrobials through the exopolysaccharide matrix and different physiological activity which is caused by the hypoxic environment [51]. *P. aeruginosa* utilises its quorum sensing (QS) molecules, such as *N*-acyl homoserine lactones (HSLs) and alkyl quinolone (AQ), as part of its major cell–cell signalling. This QS system regulates expression of virulence factors, including the formation of biofilms [52,53] and the production of pyocyanin [54], proteases [55], and rhamnolipids [54,56].

*P. aeruginosa* adapts to its environment and overcomes many challenges, such as osmotic stress [57], competition from other organisms [58], decreased nutrient availability [59], antibiotics [60], and oxidative stress [61], to survive in the CF airways. Genes associated with QS, iron acquisition, phenazine biosynthesis, and multidrug efflux were shown to have mutated to promote survival and progression to chronic infection [62]. Chronic colonisation is associated with genotypic and phenotypic changes, such as reduced QS [63], metabolism [64], and motility [65] and overproduction of alginate [66] and increased antibiotic resistance [67,68]. During the chronic colonisation stage, *P. aeruginosa* changes from a non-mucoid motile phenotype to a mucoid biofilm former that is capable of evading host responses and antibiotics, at which point it becomes very difficult to eradicate.

MDR *P. aeruginosa* pose a number of therapeutic and diagnostic challenges in the CF population. As stated by the World Health Organisation (WHO), new antipseudomonal antibiotics are needed, but the introduction of new antibiotics is often closely followed by the emergence of resistance. A better understanding of the drivers and mechanisms of resistance is needed. There is a growing body of evidence showing that interactions within and among species in the microbiome can alter the virulence and antibiotic resistance of *P. aeruginosa*. These interactions may shape the evolution, virulence, and antimicrobial resistance (AMR) of *P. aeruginosa.* Increased understanding of community dynamics in the CF microbiome could provide new opportunities for therapeutic interventions. Understanding more about how chronic *P. aeruginosa* interacts with the other airway community members may provide insights into novel therapeutic approaches for these patients, and it may be possible to suppress *P. aeruginosa* indirectly by manipulating biologically relevant interactions.

## 3. The CF Airway Microbiome

There is a diverse array of bacteria, fungi, and viruses present in the airways, many of which have not previously been associated with the CF [69,70]. NGS has improved the detection of previously under-identified genera, such as *Streptococcus*, and has identified anaerobic genera such as *Prevotella* and *Veillonella* as not only common in the CF airway but highly abundant [71,72,73]. Microbiome studies corroborate culture data showing that species such as *S. aureus, H. influenzae*, and *Streptococcus* are dominant in early childhood, and pathogens, such as *P. aeruginosa*, become dominant in adulthood [74,75,76]. The diversity of the CF airway microbiome decreases with age and pathogen dominance [74,77,78,79].

The CF airway is known to be a hypoxic microenvironment, and it is becoming increasingly evident that anaerobes are important in CF disease [76,77,80,81]. Anaerobes such as *Streptococcus*, *Haemophilus, Veillonella, Neisseria, Prevotella*, *Fusobacterium, Propionibacterium, Actinomyces, Gemella*, and *Granulicatella* are commonly detected in the CF airway using culture-independent techniques, but anaerobes are rarely detected using standard culture-based diagnostics [80,82,83,84]. In early childhood, anaerobic bacteria are in significantly lower abundance in children with CF when compared with children without CF [85]. A higher relative abundance of anaerobes has recently been linked to milder disease in CF, with better nutritional status, pancreatic sufficiency, and better lung function. Reductions in anaerobes were associated with the emergence of pathogenic bacteria genera, such as *Pseudomonas* and *Stenotrophomonas* [79,81]. While there is mounting evidence supporting anaerobes as beneficial in CF, little is understood about how anaerobes may behave in the host. Anaerobes in the airway can produce short chain fatty acids (SCFAs) by fermentation of mucins in the CF airway [86]. SCFAs have been detected in CF sputum at a mean concentration of 1.99 mM and were positively correlated with sputum neutrophil counts [87]. SCFAs have been shown to either increase or decrease *P. aeruginosa* growth in a concentration-dependent manner and stimulate neutrophil chemotactic agents and production of interleukin-8 (IL-8) in CF epithelial cells [87,88]. *P. aeruginosa* cannot derive SCFAs from mucins but can utilise those formed by other microbiota members, suggesting a potential cross-feeding mechanism in the CF airway microbiome that requires further exploration.

Linking microbial community composition to measures of disease severity such as exacerbation and lung function is vital to establishing the significance of the microbiome in CF lung disease. Community composition fluctuations were monitored in a 10-year study of 111 CF patients [89]. Changes to the microbiome during exacerbation were shown to be largely dependent on community composition and diversity at baseline, with particular genera such as *Pseudomonas* and *Gemella* acting as important drivers of change [78]. Alternatively, other studies have shown there to be little change in microbial community composition in patients during exacerbation and when clinically stable [90,91]. While the link between exacerbation and microbial community composition is not clear, poor lung function is clearly correlated with reduced microbiome diversity, as has been reported in a number of studies [92,93,94].

### Antibiotics and the CF Airway Microbiome

Antibiotic treatment is a cornerstone of CF care. Antibiotics are prescribed based on the resistance profile of microorganisms cultured from sputum during exacerbation. Unfortunately, clinical responses do not often correlate with in vitro sensitivity tests, and so treatments are often unsuccessful [29,30].

Antibiotics and lung function have been found to be the main drivers of changes in microbiome composition [95]. In a study of 126 sputum samples from 6 age-matched male CF patients, spanning nearly a decade, antibiotics, rather than age or lung function, were the primary driver of decreasing diversity [96]. Despite this, communities demonstrated both short-term and long-term resilience after antibiotic-driven changes. Corroborating these findings, another study revealed that any changes in the CF airway microbiota caused by antibiotics were transient and that taxa linked to CF infection were still detected post-antibiotic treatment [97]. Practices around antistaphylococcal prophylaxis in children with CF differs from country to country, with prophylaxis currently recommended in the United Kingdom [98] and advised against in the United States [99], while in Ireland there is no consensus, with individual clinics differing in their approach. In a prospective observational study of 32 infants with CF, microbial diversity was lower in those receiving amoxicillin-clavulanate anti-staphylococcal prophylaxis [100]. In contrast, another study recently published found that prophylactic antistaphylococcal flucloxacillin treatment did not perturb the early CF airway microbiome or significantly reduce the relative abundance of *Staphylococcus* [85]. These contrasting outcomes call into question the benefits of prophylactic antistaphylococcal treatment in children with CF [101,102,103]. Some microbiome studies have revealed that the use of antibiotics such as azithromycin significantly reduced the relative abundance of the *Pseudomonas* genus [85], while other studies report that *P. aeruginosa* abundance was either not affected by antibiotics [91] or actually increased while non-pseudomonal taxa decreased during antibiotic treatment [104]. Anaerobes were more abundant in CF patients who took fewer antibiotics during their lifetime [89], and mean short chain fatty acid (SCFA) concentrations were significantly lower after antibiotic treatments [87], suggesting a reduction in anaerobes after antibiotic treatment. A very interesting study published in 2017 monitored the microbiome in 24 CF patients receiving Aztreonam lysine for inhalation (AZLI) for chronic *P. aeruginosa* infection [105]. While no significant changes were noted in alpha or beta diversity following AZLI, specific sub-populations of organisms, *Prevotella* and *Granulicatella*, declined. Perhaps most interesting was that patients with high *Staphylococcus* and anaerobic organisms, *Prevotella* and *Fusobacterium*, responded less favourably to therapy. This suggests that certain species in the microbiome may determine the success of antipseudomonal therapy. This was one of the first published indications that polymicrobial interactions may influence antimicrobial therapy success in CF.

Studies have reported that the impact of antibiotics on the airway microbiome is transient and that baseline composition returns after 1 week [106] to 1-month post-treatment [94]. Many of these studies differ in the antibiotic used, dosage (if disclosed), oral versus intravenous delivery, severity of infection requiring treatment, and patient age groups, and thus are likely not comparable. Large, multicentre, prospective controlled studies are required to make an ultimate statement on the impact of antibiotics on the CF airway microbiome. The first microbiome-based, interventional clinical trial called CFMATTERS (clinicaltrials.gov: NCT02526004; www.cfmatters.eu, accessed 1 July 2021) began in 2017 aims to determine whether targeting nonclassical (e.g., anaerobic) species with antibiotics will improve CF outcomes.

## 4. Polymicrobial Interactions with *P. aeruginosa* in the CF Airway Microbiome

The complex network of microorganisms in the CF airway microbiome have been shown to interact with each other and their environment, and this has the potential to influence clinical outcomes. *P. aeruginosa* colonisation changes the surrounding microbiome, but how diverse members of the community interact in general is not well understood. Some studies have begun to shed light on the polymicrobial interactions taking place among these bacteria (Table 1). These studies provide evidence that inter-species, cross-species, cross-genera, and cross-Kingdom interactions can influence not only pathogen virulence and host responses but also resistance and tolerance to antibiotics. The impact of these interactions in CF airway disease are yet mostly unexplored.

During co-infection, *P. aeruginosa* remains prevalent and dominant, due to its great genomic plasticity, high virulence, and its ability to form biofilm. The pathogenicity of *P. aeruginosa* can be directly shaped by interactions with other species [107,108,109,110]. In vitro studies have been conducted on *P. aeruginosa* co-colonising and interacting with *S. aureus*, *Streptococci*, *B. cepacia* complex, *A. fumigatus, C. albicans*, and *S. maltophilia* (Table 1). Furthermore, studies have shown that these interactions can be synergistic and/or antagonistic, shaping *P. aeruginosa* virulence, thus enhancing its pathogenicity and persistence [69,111,112]. For a more comprehensive review of polymicrobial interactions impacting virulence in CF, there are some excellent reviews already published [6,69,113]. The present review will focus on the impact of polymicrobial interactions on *P. aeruginosa* resistance/tolerance to antibiotics and the potential implications this has on therapeutic approaches in CF.

### 4.1. P. aeruginosa and S. aureus Interactions

One of the most studied co-infections to date in this field is *P. aeruginosa* and *S. aureus*. *S. aureus* is often the first bacteria detected in people with CF and is more common in childhood [148,149,150]. One-third of patients with CF are estimated to be co-infected with *S. aureus* and *P. aeruginosa*, and their interactions have been linked to a more rapid lung function decline [151,152]. A 13-year study recently demonstrated an increased trend in prevalence of *S. aureus* and *P. aeruginosa* coinfections, from 30.6% in 2004 to 50.7% in 2016. In contrast with previous understanding, largely based on the US CFF reports, *P. aeruginosa* infections did not overtake *S. aureus* infections. Instead, CF patients were co-colonised with both bacteria at the same time [110].

Several studies have been conducted in this area, and there have been reports of competitive and cooperative *P. aeruginosa*–*S. aureus *interactions (Table 1). *P. aeruginosa* has been shown to compete with *S. aureus* by expressing exoproducts, such as elastase [120] and rhamnolipids [153,154]. Other studies have shown a synergistic interaction between these pathogens, with *S. aureus* stimulating the production of staphyloxanthin pigment [155], and these synergistic interactions have been linked to decreased pulmonary function [17]. A recent study has shed some light on the potential reason for the disparity in these studies. Sixty-four *S. aureus* clinical isolates from CF patients were tested for their ability to interact with *P. aeruginosa* and ranged from highly sensitive to completely tolerant to *P. aeruginosa* [156]. Many studies have now reported that long-term co-adaption of these two pathogens results in a more commensal, cooperative relationship [154,157,158].

#### Impacts on Antibacterial Resistance

**Figure 1:**
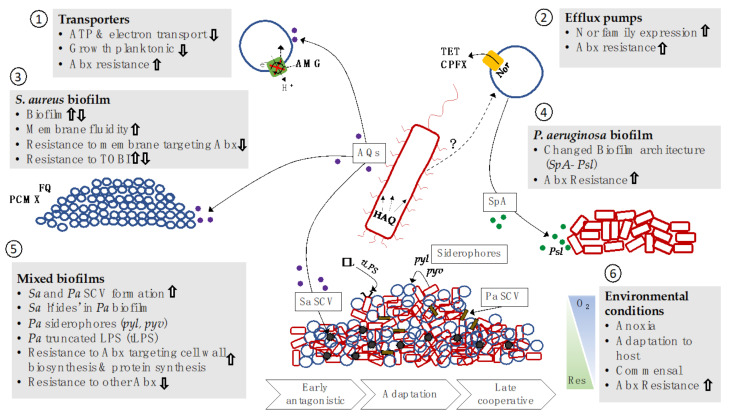
*P. aeruginosa* and *S. aureus* interactions leading to changes in antibiotic resistance. (**1**) AQs produced by *Pa* impact *Sa* planktonic growth, decreasing ATP generation and active transport and so uptake of AMG. (**2**) *Sa* Nor efflux pumps are upregulated in the presence of *Pa*, causing increased resistance to TET and CPFX. (**3**) *Pa* produced AQs, HQNO, and HAQ increase *Sa* biofilm production and membrane fluidity and decrease resistance to membrane-targeted antibiotics. Studies differ in increase/decreased resistance to TOBI and increase/decrease *Sa* biofilm. (**4**) *Sa*-produced *SpA* interacts with *Pa* exopolysaccharide *Psl*, changing *Pa* biofilm architecture and resistance to antibiotics. (**5**) Prolonged mixed biofilms and long-term exposure to HQNO induce *Sa* SCVs. *Pa* in mixed biofilms increases siderophore production and produces truncated LPS both linked to AMR to antibiotics targeting cell wall biosynthesis and protein synthesis but unchanged or decreased resistance to other antibiotics. (**6**) Environmental conditions such as anoxia and adaptation to host increase antibiotic resistance and commensal cooperative behaviour. *Pa* = *P. aeruginosa*, *Sa* = *S. aureus*, Abx = antibiotics, AMG = aminoglycosides, SCV = small colony variant, TET = tetracycline, CPFX = ciprofloxacin, FQ = fluoroquinolones, PCMX = chloroxylenol, βL = beta-lactam, TOBI = tobramycin, SpA = Staphylococcal protein A, Psl = biofilm exopolysaccharide, pyl = pyochelin, pyv = pyoverdine, AQ = 2-alkyl-4-(1H)-quinolones, HQNO = 4-hydroxy-2-heptylquinoline-N-oxide, HAQ = 4-hydroxy-2-alkylquinoline.

Several studies have shown that there is a bidirectional alteration in growth and resistance of *P. aeruginosa* and *S. aureus* when in co-culture (Table 2). In planktonic growth states, *P. aeruginosa* has been shown to be the dominant pathogen and to suppress *S. aureus* growth [20,155]. Some studies have found *P. aeruginosa* to suppress *S. aureus* biofilm formation [159], while others have found compounds produced by *P. aeruginosa* to increase *S. aureus* biofilm formation [160,161]. *S. aureus* completely avoided vancomycin, ampicillin, and ceftriaxone by ‘hiding’ in *P. aeruginosa* biofilms; however, the mixed biofilms were found to be more susceptible to the broad-spectrum antibiotics ciprofloxacin and aminoglycosides [162] (Figure 1). Many of these studies have focused on interactions between reference strains, with PA01 or PA14 commonly outcompeting *S. aureus* isolates. A study published in 2014 reported that early adapted strains of *P. aeruginosa* in a chronically colonised CF patient outcompeted *S. aureus*, while later-adapted strains showed commensal-like interactions [157]. Corroborated by another study in 2016, *P. aeruginosa* and *S. aureus* were shown to have a proto-cooperative relationship when human-adapted strains were studied [158]. Radlinski et al. (2017) also reported strain-dependent differences in the production of several factors by *P. aeruginosa*–*S. aureus* biofilms linked to antimicrobial resistance [154]. Pathogens are known to adapt to the host environment during chronic infections; therefore, testing reference strains alongside clinical isolates is extremely important in polymicrobial communication studies.

Efflux of antibiotics from cells via transport pumps is a well-known mechanism of AMR, and several antibiotic pumps belonging to the Nor family (*tet38*, *norA*, and *norC*) were upregulated in *S. aureus* during co-cultures and exposure to *P. aeruginosa* supernatants, leading to an increase in antibiotic resistance of *S. aureus* to tetracycline and ciprofloxacin [27].

*P. aeruginosa* produces several AQ small molecules involved in QS. The AQ 4-hydroxy-2-heptylquinoline-*N*-oxide (HQNO) has been shown to suppress *S. aureus* planktonic growth, while transiently protecting it from killing by aminoglycosides [20] (Figure 1). The authors hypothesize that this may be directly related to the ability of HQNO to inhibit *S. aureus* electron transport. HQNO is known to inhibit electron transport through cytochrome b in *S. aureus*, resulting in decreased ATP generation, a shift to fermentative metabolism, and decreased active transport, which is required for uptake of AMG [159]. Another group have also recently found that HQNO induces multidrug tolerance in *S. aureus* via respiratory inhibition and reducing cellular ATP [154]. In contrast, HQNO in supernatants of *P. aeruginosa* was found to be responsible for increasing preformed *S. aureus* biofilm sensitivity to several antimicrobial agents, including fluoroquinolones and the antiseptic, chloroxylenol [163]. HQNO has been shown to increase *S. aureus* biofilm formation, and long-term exposure was shown to induce formation of difficult to detect, slow-growing mutants of *S. aureus* known as small colony variants (SCVs) [160]. SCVs are known to aid persistence inside viable human cells and to be resistant to several antibiotics [47,164,165]. A second AQ, 4-hydroxy-2-alkylquinoline (HAQ), is an intracellular QS molecule produced by *P. aeruginosa*, and its expression was increased in a human-adapted *P. aeruginosa* isolate and upon interaction with *S. aureus* conveying resistance to tobramycin [158]. A third AQ, *Pseudomonas* Quinolone Signal (PQS), has been shown to be positively correlated with *S. aureus* biofilm formation [161]. *P. aeruginosa* mutants deficient in PQS and HQNO were less capable of stimulating biofilm formation by *S. aureus* than the wild type. It is evident that QS molecules are important in inducing resistance to several antimicrobials via their influence on electron transport and biofilm formation.

Siderophores are high-affinity iron-chelating compounds involved in transporting iron across microbial membranes. Most bacteria and fungi produce siderophores altruistically, and other species can benefit from their siderophore production. Increased siderophore production, pyochelin and pyoverdin, by *P. aeruginosa* in co-biofilms was observed to be linked to increased antibiotic tolerance [47]. In a study of 100 *P. aeruginosa* clinical isolates, a correlation between siderophore production and AMR emerged [166]. No mechanism for this siderophore-directed resistance has been elucidated.

Exoproducts of *P. aeruginosa* such as LasA endopeptidase and rhamnolipids have been shown to increase killing of *S. aureus* by vancomycin and tobramycin, respectively [154]. Rhamnolipids are glycolipid surface-active molecules with several virulence-related activities in *P. aeruginosa*, and their biosynthetic pathway is metabolically linked to numerous bacterial products, such as alginate, LPS, and HAQ [167].

Descriptions of the other side of this relationship are sparse. *P. aeruginosa* SCVs are also selected for in co-cultures with *S. aureus* [157] (Figure 1). The staphylococcal protein A (*SpA)* produced by *S. aureus* has been shown to bind to the *P. aeruginosa* EPS, *Psl*, modifying biofilm architecture and increasing resistance of *P. aeruginosa* to inhaled tobramycin [28]. Another recent study has shown bi-directional changes in resistance of both *P. aeruginosa* and *S. aureus* in mixed biofilms [162]. *P. aeruginosa* evolved for 150 generations in the presence of *S. aureus* and became more resistant to ß-lactam antibiotics but not ciprofloxacin or polymyxin due to a loss of a glycosyltransferase, *wbpL_PA14_*, involved in the biosynthesis of polysaccharide antigen and the O-specific antigen (OSA). The authors conclude that the production of a truncated LPS devoid of OSA during adaptation to *S. aureus* led to increased resistance of *P. aeruginosa* to ß-lactam antibiotics [112]. These findings suggest that *P. aeruginosa* in mixed biofilms is more resistant to antibiotics targeting cell wall biosynthesis and protein synthesis but that other resistance mechanisms may be less affected.

### 4.2. P. aeruginosa and S. Maltophilia Interactions

*S. maltophilia* is an opportunistic nosocomial Gram-negative bacterium and is inherently MDR. It has been linked to higher mortality rates and lung transplantations in CF patients [173]. *S. maltophilia* is present in roughly 8% of CF patients in Europe [174]. The frequency of co-colonisation with *P. aeruginosa* and *S. maltophilia* in CF patients ranges between 10% and 60% [175,176,177].

Only a small number of studies have examined the interactions of *S. maltophilia* and *P. aeruginosa* and the effect co-colonisation has on both bacteria and the host. *S. maltophilia* abundance increases during co-infection with *P. aeruginosa*, possibly due to the protection *P. aeruginosa* biofilms afford to *S. maltophilia*, suggesting *S. maltophilia* benefits from co-infection [50]. Nas et al. (2019) showed that *S. maltophilia* encodes a type IV VirB/D4 secretion system (T4SS) that promotes competition between other bacterial species (69). This T4SS system has a role in the interplay between the two organisms: it enhances *S. maltophilia* growth while reducing *P. aeruginosa* growth [178] (Table 1).

*S. maltophilia* also influences biofilm formation by some *P. aeruginosa* strains by reducing EPS production and thus compromising biofilm attachment to the epithelial cell surface [18]. These findings suggest that *S. maltophilia* in a mixed co-infection may benefit from increased abundance and growth while disrupting the biofilm formation of *P. aeruginosa*.

#### Impacts on Antibacterial Resistance

In a mixed biofilm, *S. maltophilia* increases *P. aeruginosa* biomass and indirectly protects *S. maltophilia* from tobramycin activity [18] Table 2. Kataoka et al. (2003) showed *S. maltophilia* provides passive resistance to *P. aeruginosa* by producing β-lactamases, allowing *P. aeruginosa* to grow in the presence of imipenem and ceftazidime [168], suggesting *S. maltophilia* can protect *P. aeruginosa* from eradication in the host.

When grown in mixed biofilm with *S. maltophilia*, *P. aeruginosa* over-expressed alkaline protease (AprA) and alginate, while the QS-related *rhlR* and *lasI* genes were downregulated [18]. Moreover, the downregulation or inactivation of lasR was previously shown to increase *P. aeruginosa* resistance to ceftazidime [63]. It was also shown that mutation of PA1396, a sensor kinase in *P. aeruginosa*, or addition of the diffusible signal factor (DSF) by *S. maltophilia* to *P. aeruginosa* resulted in increased levels of proteins implicated in resistance to cationic antimicrobial peptides; this effect was associated with increased tolerance to polymyxins [111]. Collectively, these findings suggest that *S. maltophilia* is capable of changing the virulence and antibiotic resistance of *P. aeruginosa*.

### 4.3. Anaerobes Impact P. aeruginosa Antibiotic Resistance

Anaerobic bacteria are not traditionally tested for in medical laboratories and therefore have not been commonly reported in CF. However, it is well established that the CF airways are hypoxic and that hypoxia is greater in later and more severe CF. Employing culture-independent approaches, anaerobes have now been shown to be key members of the CF airway microbiome. Up to 91.1% of CF sputa have been shown to be positive for at least one anaerobic bacterial species [179]. Anaerobes are increasingly dominant in patients with better lung function [95], and several studies have reported the negative impact of antibiotics on anaerobic populations in the CF airway [87,89,104,105]. The general consensus forming is that anaerobes may be beneficial in the airway microbiome.

Anaerobes have been shown to provide ‘passive resistance’ or indirect resistance to surrounding members of the community by producing extracellular metabolites or enzymes capable of degrading antibiotics in the vicinity. Species in the strictly anaerobic genus *Prevotella* are now commonly detected in the CF airways and represent around 70% of the anaerobes in the CF airway microbiome [180,181]. A study analysing the antibiotic resistance of 107 *Prevotella* isolates from CF and non-CF patients found that 50% of isolates produced ß-lactamases. In another study by the same group, up to 76% of *Prevotella* isolates cultured from adults and young children with CF produced extended-spectrum ß-lactamases which correlated with higher MICs to ß-lactam antibiotics [169]. They performed a co-culture experiment and a ß-lactamase-positive *Prevotella* protected *P. aeruginosa* from ß-lactam activity. This highlighted the real prospect of ‘emerging’ anaerobic bacteria being key roadblocks in the treatment of CF airway infections.

Several other anaerobic genera are now known to colonise the CF airway, including *Veillonella, Porphyromonas, Streptococci*, and *Fusobacterium*. While interactions between species in these genera and *P. aeruginosa* have been studied and reported changes in virulence [122,126,182] few studies have explored the ability of the interactions to change *P. aeruginosa* antibiotic tolerance.

In a multispecies microaerophilic biofilm model of species commonly co-colonising the CF lung—*P. aeruginosa*, *S. aureus*, *Streptococcus anginosus*, *Achromobacter xylosoxidans*, *Rothia mucilaginosa*, and *Gemella haemolysan—*there was no difference in antibiotic susceptibility when compared with single species biofilms. Not only did adding anaerobic species *P. melaninogenica*, *Veillonella parvula*, and *Fusobacterium nucleatum* to the multispecies biofilm not influence antibiotic susceptibility, but they also found that the common CF bacteria in multispecies biofilms had similar susceptibility to antibiotics as their single-species biofilm correlates [183]. While this model is possibly one of the most sophisticated airway-relevant polymicrobial biofilm models published, it is still quite simplified and AMR profiles of the anaerobic bacteria included were not reported, specifically whether the *P. melaninogenica* strain was a ß-lactamase producer was not disclosed. With the full appreciation of the complexity involved in establishing in vitro multispecies models of the microbiome, further developments to include a variety of strains that would be representative of the types of resistant strains encountered in CF could yield very powerful results.

### 4.4. P. aeruginosa and Fungal Interactions

Although much attention is given to the bacteria that colonise the CF airway, fungi are also important in CF lung infections. The most commonly isolated fungi from the CF airways include *Aspergillus fumigatus, Candida albicans, Scedosporium* spp., *Pneumocystis jirovecii*, and *Exophiala dermatitidis*.

#### 4.4.1. *P. aeruginosa* Interacts with *A. fumigatus*

*A. fumigatus* is isolated from 11% to 60% of patients [184,185,186]. Generally intermittent or persistent *A. fumigatus* colonisation is not treated unless allergic bronchopulmonary aspergillosis (ABPA) is confirmed. However, it was shown that administration of itraconazole to non-ABPA CF population with *Aspergillus* colonisation resulted in reducing *Aspergillus* bioburden, stabilising lung function while reducing exacerbations, and improving quality of life [187], suggesting a pathogenic role for *A. fumigatus* in CF.

The prevalence of *A. fumigatus* and *P. aeruginosa* co-colonisation in patients with CF is 15% [109]; however, *P. aeruginosa* colonises 54% of CF patients with persistent *A. fumigatus* infection [116]. Co-colonisation with these two microorganisms results in reduced lung function, increased number of hospitalisations, respiratory exacerbations, and increased usage of antimicrobials relative to colonisation with either pathogen alone [16].

Complex antagonistic and synergistic interactions have been reported between *A. fumigatus* and *P. aeruginosa. P. aeruginosa* has been reported to inhibit *A. fumigatus* growth using a number of mechanisms, such as phenazine production [135,136], iron competition [137,138] development of SCVs [139], metacaspases [132], and expression of QS molecules [135,140,141,142]. *A. fumigatus* also reduces *P. aeruginosa* growth, and this is largely due to the production of gliotoxin [21,134]. *P. aeruginosa* and *A. fumigatus* interactions contribute to an altered pro-inflammatory response and increased pathogenicity, which could have significant implications for the treatment of this mixed infection [133,134].

#### 4.4.2. Impact on Antimicrobial Resistance

Bacterial and fungal cells embedded in mixed microbial extracellular matrix are highly resistant to antimicrobials. Manavathu et al. (2014) performed in vitro antimicrobial susceptibility studies in monomicrobial and polymicrobial biofilms of *A. fumigatus* and *P. aeruginosa* [21]. Polymicrobial biofilms were significantly less susceptible to cefepime, suggesting that *A. fumigatus* may be changing the resistance profile of *P. aeruginosa* by enhancing its biofilm make-up.

Margarlit et al. (2020) demonstrated that the *A. fumigatus* secretome alters the proteome of *P. aeruginosa* and, of particular interest, increases the levels of proteins involved in efflux pumps and outer membrane proteins (OMPs) [170]. OMPs regulate the influx and efflux of nutrients and toxic compounds from the cell. In this study, the abundance of the OMP OprM was significantly increased; OprM forms the ejection component of the MexAB-OprM efflux system and is responsible for β-lactam and quinolone resistance in *P. aeruginosa* [188,189].

### 4.5. P. aeruginosa and C. albicans Interactions

*C. albicans* is an opportunistic pathogenic yeast and is one of the most commonly isolated microorganisms from CF sputum, with approximately 75% prevalence [184]. *Candida* spp. are generally considered colonisers of the upper respiratory tract and usually regarded as not clinically relevant. However, there are now several studies showing evidence of interactions and interplay between *P. aeruginosa* and *C. albicans* that may influence antibiotic resistance and host response to infection.

*P. aeruginosa* inhibits *C. albicans* growth in the host [190,191], and *P. aeruginosa*-expressed LPS [143] and the QS molecules 3-oxo-C12HSL [145] and 2-heptyl-3-hydroxyl-4-quinolone [192] have been shown to inhibit *C. albicans* biofilm formation and hyphal development. In particular, 3-oxo-C12HSL can even reverse the switch from yeast to hyphal growth in *C. albicans* and is important for adherence of bacterial cells to *C. albicans* [144]. Competition between these microorganisms may be strain- and growth-state dependent. Hogan and Kolter (2002) showed some strains of *P. aeruginosa* are cytotoxic to the filamentous form of *C. albicans* but are unable to attach to or kill *C. albicans* yeast cells [193].

The interaction between these two organisms has been shown to be bidirectional. Farnesol, a virulence factor secreted by *C. albicans*, leads to decreased PQS production in *P. aeruginosa*, resulting in a reduction of the PQS-regulated virulence factor pyocyanin [146]. Farnesol also influences expression of *P. aeruginosa* virulence-related proteins including haemolysin [147] and inhibits swarming motility [144]. *P. aeruginosa* and *C. albicans* are clearly capable of interacting, and this has an impact on their virulence mechanisms.

#### Impact on Antimicrobial Resistance

*C. albicans* enhanced *P. aeruginosa* ECM production, consisting of proteins, polysaccharides, and nucleic acids, through increased expression of the alginate genes *AlgU* and *mucA* [171]. Alam et al. (2019) showed *C. albicans* can enhance meropenem tolerance of *P. aeruginosa* in a dual-species biofilm [172]. They concluded that fungal mannan and glucan secreted into the ECM of a dual species biofilm of *P. aeruginosa* and *C. albicans* increases *P. aeruginosa* tolerance to meropenem. These studies highlight that cross-Kingdom co-infections can have a direct impact on antibiotic tolerance of pathogenic bacteria.

### 4.6. Other Fungi

*Scedosporium apiospermum* species complex are the second most prevalent filamentous fungi cultured in CF after *A. fumigatus* [194]. A few studies to date have examined the interplay between *P. aeruginosa* and *Scedosporium*. *P. aeruginosa* was shown to inhibit *S. aurantiacum* in vitro [195]. Chen et al. (2017) used florescence microscopy to reveal poorly formed hyphae when cultured with *P. aeruginosa*, with non-mucoid strains more commonly having an inhibitory effect [195]. *P. aerugionosa* inhibited the growth *S. aurantiacum*, which was shown to be mediated by the production of biologically active metabolites. Biofilm formation and colonisation of fungal hyphae by *P. aeruginosa* were also important for *S. aurantiacum* growth inhibition [196]. Homa et al. (2019) also confirmed that *P. aeruginosa* is inhibitory towards *Scedosporium* through direct cell contact but also showed growth-promoting effects revealing that *P. aeruginosa* was able to enhance the growth of *Scedosporia* via VOCs [194]. These studies show that *Scedosporium *have been shown to alter the virulence of *P. aerugionosa*; however, fungi, other than *A. fumigatus* and *C. albicans*, have not been tested for their ability to increase bacterial tolerance to antibiotics.

## 5. Direct Transfer of Resistance in the CF Airway Microbiome

The co-colonisation of the CF airway by several microbial species provides vast opportunity for transfer of mobile genetic elements carrying ARGs. Crosstalk between members of the CF airway microbial community and horizontal gene transfer shape the microbial community for adaptation to the host environment. Whole genome shotgun sequencing has been employed in a number of studies to date to study the metabolic capabilities, virulence mechanisms, and resistance profiles or ‘resistome’ of the CF microbial community.

The ARG repertoire appears to be consistent among patients of similar disease status and not related to pre-exposure to antibiotics. A study monitoring 22 CF patients with more severe lung disease over 15 months reported that despite differing exposures to antibiotics among their patient cohort, the same repertoire of ARGs was present among almost all patients [97]. Other studies have also reported that the variety of ARGs detected in the diseased airway resistome was independent of prior exposure to antibiotics [197]. While the taxa present differed between patients, the ARGs present in the community were less variable. The resistome was shown to be different among patients with mild and severe disease. Adults with CF that had poorer lung function and more severe disease had increased abundance of efflux-mediated antibiotic resistance genes [198]. Ultra-deep metagenomic shotgun sequencing of 85 individuals with chronic respiratory disease revealed a ‘core’ airway resistome dominated by macrolide, ß-lactam, fluoroquinolone, and tetracycline resistance genes [197]. They delved further to explore the source of the ARGs and found that *Streptococcus* and *Actinomyces* were the main microbial reservoirs of macrolide resistance.

Bacteriophages are vehicles for transfer of mobile genetic elements between bacteria in the microbiome. DNA viral sequencing of CF sputum has revealed a CF phageome distinct from the phageome of people without CF [199]. Studies investigating the CF airway virome have revealed widespread genomic rearrangements and phage activity in the CF airway during periods of exacerbation and intravenous antibiotic treatment [200]. Important CF pathogens including, *P. aeruginosa, S. aureus*, and *B. cenocepacia*, have been shown to contain antimicrobial inducible prophages in their accessory genome [201,202]. Metagenomic studies of CF airway viral communities have revealed the presence of ARGs in bacteriophage. One such study identified a large number of resistant genes on mobile elements in antibiotic naive samples from infants with CF [203], suggesting that resistant genes are present in the community before antibiotic exposure. Up to 32.9% of ß-lactamase genes detected in this study were on mobile genetic elements, highlighting the risk for ARG transfer within the CF microbiome. Another study of 130 nasal swabs from 26 infants with CF found that the CF airway microbiome of antibiotic naïve patients had a large number of ARGs, including mobile elements [203].

The presence of resistance mechanisms, in particular ARGs on mobile elements, in the microbiome may be more relevant than identifying specific bacteria and their resistance profiles. This area requires further large multicentre studies in order to establish the role that monitoring the resistome could play in clinical decision making.

## 6. Summary

**Figure 2:**
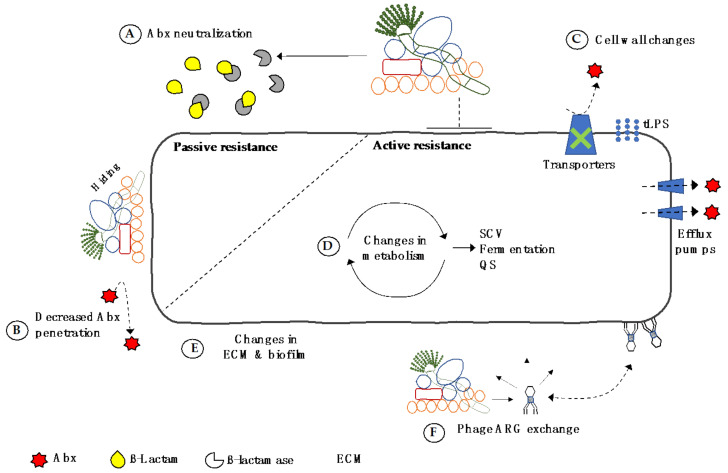
Passive and active mechanisms of antibiotic tolerance/resistance developed by *P. aeruginosa* during polymicrobial interactions. (**A**) Neutralisation of antibiotics by cleaving enzymes produced by other community members; (**B**) ‘hiding’ in multispecies biofilms reducing access of antibiotics; (**C**) changes in cell wall architecture, including truncated LPS, increased expression of efflux pumps, or reduced activity of membrane transporters; (**D**) changes in metabolism resulting in alterations to growth and quorum sensing, development of SCVs, and conversion to fermentative growth; (**E**) increased or decreased production of ECM and biofilm growth; and (**F**) transfer of mobile ARGs via bacteriophage. Abx = antibiotics, ECM = extracellular matrix, SCV = small colony variant, QS = quorum sensing, tLPS = truncated lipopolysaccharide, ARGs = antibiotic resistant genes.

Facing the very real threat of AMR and MDR infections limiting our use of antibiotics in the future, research must focus on understanding all mechanisms by which microorganisms develop resistance. *P. aeruginosa* is one of the microorganisms listed by the World Health Organisation (WHO) for which new antibiotics are urgently required due to the high levels of MDR in this pathogen. This review has collated extensive evidence that *P. aeruginosa* is less susceptible to antibiotics when co-cultured with several microorganisms of the airway microbiome, such as *S. aureus, S. maltophilia, Prevotella*, and *A. fumigatus*. The mechanisms of polymicrobial antibiotic tolerance can be broadly divided into passive and active categories (Figure 2). Passive mechanisms include pathogens benefiting from the production of antibiotic cleaving enzymes produced by other members of the microbial community and ‘hiding’ in polymicrobial biofilms. Active mechanisms of resistance involve more direct changes in *P. aeruginosa*, including increased expression of EPS, increased biofilm formation, changes in membrane architecture (increased expression of efflux pumps, truncated LPS, and reduced activity of membrane transporters), changes in metabolism, and the acquisition of ARGs via horizontal transfer of mobile genetic elements.

Two studies have modelled multispecies biofilms in vitro, both containing anaerobes, and both reported that *P. aeruginosa* was not more resistant to antibiotics in these models than when cultured alone [123,183]. Tavernier et al. (2017) created a multispecies biofilm model composed of *P. aeruginosa*, *S. aureus*, and *S. anginosis* and found that *P. aeruginosa* was not more susceptible or tolerant to antibiotics when in a mixed species biofilm [123]. These studies model simplistic polymicrobial communities but contradict all other findings from co-culture studies reviewed here. Further studies in this area are required, and there is a significant requirement for developing representative in vitro multispecies models to further explore the impacts of inter-species, cross-species, and cross-Kingdom interactions on the resistance of important pathogens. The ability of microorganisms within a polymicrobial community such as the CF airway microbiome to share ARGs and antibiotic-cleaving enzymes and to encourage tolerance at a community level may explain why in vitro sensitivity tests do not correlate with clinical outcomes in CF.

The evidence is undeniably complex, and with many studies reporting contradictory findings, the overall picture is difficult to decipher. Mode of microbial growth, strain type (adapted/non-adapted), duration of mixed biofilm growth, antibiotic mode of action, and environmental conditions under which all are tested are all significant factors to consider when interpreting the outcome of mixed species relationships. Collectively, the findings from studies to date suggest that *P. aeruginosa* mixed infections may be more resistant to antibiotics and more persistent in the host.

## 7. Conclusions

Studies that report increased resistance due to polymicrobial interactions suggest combination antibiotic therapy; however, this is already practiced in CF therapy, and a recent meta-analysis found that there was no significant difference between single versus combination anti-pseudomonal antibiotic therapy on outcomes such as lung function symptom scores, adverse effects, and microbial burden reduction [204]. With the limited development of new antibiotics, one avenue in the future development of alternative antimicrobial therapies could target polymicrobial interactions that promote resistance or antibiotic tolerance.

The QS molecule HQNO produced by *P. aeruginosa* is known to cause respiratory deficiency in *S. aureus* when in co-culture, and respiratory deficient *S. aureus* is hypersensitive to the plant alkaloid tomatidine (TO). Boulanger et al. (2015) tested to see if this polymicrobial interaction could in fact make *S. aureus* more susceptible to killing by tobramycin [205]. TO increased the killing of *S. aureus* by 3.4 log10 CFU/mL in comparison with that observed in a co-culture without TO. Employing several mutant strains, this increased killing was shown to be linked to lasR rhlR, pqsA, pqsL, or lasA, and supplementing the pqsl mutant with HQNO restored bactericidal activity. The bactericidal activity of TO was also observed against a tobramycin-resistant methicillin-resistant *S. aureus* (MRSA) and *P. aeruginosa* in co-culture. HQNO (0.39 to 1.24µg/g) has been detected in the sputa of CF patients colonized with *P. aeruginosa* in a bioburden-dependent fashion [20]. Long-term exposure of *S. aureus* to HQNO in co-culture with *P. aeruginosa* induces formation of difficult-to-detect, slow-growing, antimicrobial-resistant SCVs of *S. aureus* [160]. HQNO may also be useful as a biomarker of persistent *P. aeruginosa–S. aureus* co-infection and the development of SCVs which are much harder to treat [47,164,165]. These studies are excellent examples of how polymicrobial interactions could be manipulated to enhance treatment of mixed microbial infections.

Competition for limited iron availability in the host can be a key driver of competitive relationships within polymicrobial communities. *P. aeruginosa* produces higher levels of its siderophores, pyochelin and pyoverdin, in co-biofilms [47] and increased siderophore production has been linked with emergence of AMR [47,166]. Siderophore–antibiotic conjugates that essentially hijack the iron-mediated active transport systems of microorganisms have been designed and enhance uptake of antibiotics. Cefiderocol (S-649266), a cephalosporin–catechol conjugate and the first to make it to phase III trials, has shown potent activity against *P. aeruginosa* and several other MDR Gram-negatives [206,207]. Some bacteria naturally perform this ‘*Trojan horse strategy*’ by producing their own siderophore–antibiotic conjugates, sideromycins, to compete with other species in the community. These siderophore–antibiotic conjugates could be important therapies in polymicrobial infections, where iron uptake systems of *P. aeruginosa* have been shown to be particularly active due to increased competition for limited iron sources in the host environment.

The CF airway is host to a large population of anaerobic bacteria, and these bacteria produce SCFAs such as butyrate, propionate, and acetate [86,87] which among other functions are part of a complex cross-feeding network in microbial communities. SCFAs have been shown to either increase or decrease *P. aeruginosa* growth in a concentration-dependent manner and stimulate neutrophil chemotactic agents and production of interleukin-8 (IL-8) in CF epithelial cells [87,88]. SCFAs are well-known to have anti-inflammatory properties in the gut and to promote gut epithelial health; however, studies of their therapeutic potential in gut disease have resulted in disparate outcomes [208]. SCFAs have been shown to induce excessive production of IL-8 in CF epithelium cell lines [88], and while using SCFAs therapeutically in CF is unlikely, disrupting cross-feeding mechanisms may reduce *P. aeruginosa* fitness. *P. aeruginosa* cannot derive SCFAs from mucins but uses those formed by other microbiota members, and disrupting or manipulating cross-feeding mechanisms between community members as a therapeutic approach warrants further study.

A perplexing phenomenon in CF and other chronic respiratory diseases is the disparity between in vitro antimicrobial sensitivity of cultured bacteria and clinical response to antibiotic therapy [29,30]. Many traits of polymicrobial community behaviour discussed in this review could contribute to this discrepancy. In particular, the production of antibiotic cleaving enzymes by community members unculturable by standard diagnostic practices go completely undetected and are not considered when planning therapy. The main reservoir of macrolide resistance in the CF airway microbiome has been shown to be in *Streptococcus* and *Actinomyces* species [197], two anaerobic bacteria rarely detected by culture and traditionally not considered as significant in CF disease. Relying solely on culture-based diagnostics and only looking at the resistance profiles of the common CF pathogens in isolation is not representative of the host environment. Monitoring the CF airway resistome and focusing on the ARGs present in the microbiome as a whole may provide more clinically relevant information that clinicians can use when planning therapy. For instance, detecting ß-lactamase genes in any member of the microbiome may be a good indicator for prescribing ß-lactamase inhibitors alongside ß-lactams. Few studies have been published on the ‘resistome’ in CF, and further studies are required to assess the impact of community resistome data on prescribing and improved health outcomes.

Passive resistance and resistance/tolerance acquired due to polymicrobial interactions are understudied. As the world is on the cusp of an antibiotic resistance crisis, it is vital that we gain a keen understanding of all mechanisms by which microorganisms can avoid, tolerate, and resist antimicrobial agents.

## Figures and Tables

**Table 1:** Studies reporting microbial interactions with *P. aeruginosa*.

*Sp* Interacting with *PA*	Microbial/Host Response	Potential Implications on Disease	Ref(s).
**Gram-positives**	↑ lytic activity by PA ↓ Gram+ in vivo models	PA more toxic in co-infections with Gram+	[114]
	↑ pyocyanin production by PA	PA mechanisms of dominance	[115]
***S. aureus***	Co-infection strains less competitive than mono-infection strains	Adaptation to coexistence in the lung	[116]
	PA induces bronchial epithelial cells to produce phospholipase, sPLA2-IIA	Manipulation of host immune response, enhanced survival of PA, and killing of SA and other Gram+	[117]
	PA EPS can affect mixed species biofilm architecture	Proximity of SA and PA in mixed biofilms	[118]
	↑ PA siderophore production Lysis of SA	Iron competition LasA protease	[112] [119,120]
	PA LPS inactivation mutations ↓ production of PA LPS in anoxia	Reduced recognition by immune system, persistence Immune evasion	[112] [112]
	↑ PA swimming motility in anoxia	Reseeding of infection in lung	[17]
***S. maltophilia***	Co-colonise the CF airway	Opportunity to interact	[121]
	↓ SM growth ↑ PA biofilm	Altered virulence and persistence	[18] [111]
	↓ Adhesion of PA to CFBE	Evasion of immune system and persistence	[18]
***Streptococci* spp.**	↓ SMG growth—PA competition for iron ↓ *S. anginosus *growth and biofilm formation ↑ *Strep* spp. biofilm formation—hijacks PA EPS ↓ PA viability—Strep H_2_O_2_ production	Pathogen dominance and persistence Altered virulence and persistence Persistence Strep beneficial to host	[122] [123] [124,125] [126]
***Burkholderia cepacia complex***	↓ PA virulence factors	Altered virulence	[127]
	PA enhances Bcc virulence	Altered virulence	[128]
	↓ PA growth in vivo	Altered persistence	[129]
	Reduced growth of Bcc and PA	Competition beneficial to host?	[130]
	Co-infection ↑ inflammatory markers	Increased host inflammation	[19]
***A. fumigatus***	Co-colonise the CF airway	Opportunity to interact in airway	[131]
	Co-colonised patients—↓ lung function, ↑ hospitalisations, exacerbations and Abx usage	Poorer disease outcome	[16]
	PA SNs stimulate AF growth	Increased AF abundance in co-infections *	[131]
	Metacaspases from Pa SNs inhibit and damage AF biofilms	Reduced AF abundance in co-infections	[132]
	↑ elastase production by PA in presence of AF	More damaging pathology	[133]
	SNs from co-cultures more toxic to epithelial cells lines	More damaging pathology	[133]
	Mutually antagonistic	Competition beneficial to host?	[134]
	Gliotoxin produced by AF reduces PA biofilm	Competition beneficial to host?	[134]
	Co-infections cause altered inflammatory response	Evasion of the immune system and persistence	[134]
	PA dirhamnolipids induce AF ECM production	Inhibits AF growth and facilitates PA binding	[135]
	PA phenazines inhibit AF growth by direct contact	Reduced AF abundance in co-infections	[136]
	Subinhibitory concentrations of PA phenazines can promote AF growth	Increased AF abundance in co-infections *	[136]
	Iron competition	Reduced AF abundance in co-infections	[137,138]
	Development of PA SCVs	Reduced AF abundance in co-infections	[139]
	Expression of QS molecules	Reduced AF abundance in co-infections	[135,140,141,142]
***C. albicans***	PA expressed LPS inhibits CA biofilm formation and hyphal development	Reduced CA abundance and virulence in co-infections	[143]
	PA QS molecule, 3-oxo-C12HSL	Reduced CA abundance in co-infections	[144]
	PA 2-heptyl-3-hydroxyl-4-quinolone	Reduced CA abundance in co-infections	[145]
	CA secreted Farnesol reduces PA pyocyanin Farnesol inhibits PA haemolysin Farnesol inhibits PA swarming motility	PA less virulent	[146]
		PA less virulent	[147]
		PA less virulent	[144]
	CA secreted tyrosol inhibits PA haemolysin and protease production	PA less virulent	[147]

PA = *P. aeruginosa*, SA = *S. aureus*, SM = *S. maltophilia*, AF = *A. fumigatus*, CA = *C. albicans*, Bcc = *Burkholderia cepacia complex*, SMG = *Streptococcus milleri* group, SNs = supernatants, EPS = exopolysaccharide, LPS = lipopolysaccharide, CFBE = cystic fibrosis bronchial epithelial cells, ECM = extracellular matrix, Abx = antibiotics, QS = quorum sensing, SCVs = small colony variants. * contradictory findings.

**Table 2:** Altered resistance or tolerance to antibiotics in co-cultures with *P. aeruginosa*.

Species Interacting	Resistance (Increased/Decreased)	Antibiotic	Mechanism	Ref/s.
***S. aureus*** **(+*PA)***	Increased	Aminoglycoside (gentamicin), tetracycline	Suppressed planktonic cell growth	[155]
		Tetracycline and fluoroquinolone (ciprofloxacin)	*tet38*, *norA*, and *norC* efflux pumps belong to Nor family of pumps upregulated	[27]
		Aminoglycosides	Suppressed biofilm growth SCVs persistence and poor detection Reduced uptake due to inhibition of electron transport	[20,160]
		Vancomycin, ampicillin, and ceftriaxone	‘Hiding’ in mixed biofilm	[162]
		Glycopeptide (vancomycin)	PA HQNO PA siderophores pyocyanin and pyroverdinn Anoxia	[47]
		Aminoglycoside (tobramycin)	HAQ production by human-host adapted PA strain	[158]
		MDR tolerance	HQNO induced respiratory inhibition and reduction of cellular ATP	[154]
	Decreased	Aminoglycoside (tobramycin), glycopeptide (vancomycin)	rhamnolipids facilitate proton-motive force-independent uptake of tobramycin LasA endopeptidase potentiates lysis by vancomycin	[154]
		Fluoroquinolones, membrane targeting antimicrobials, antiseptics	Increased sensitivity of SA biofilms SA biofilm cell membranes more fluid	[163]
***P. aeruginosa*** **(+*SA)***	Unchanged	Aminoglycoside (gentamicin), tetracycline	In planktonic co-cultures	[155]
	Increased	Aminoglycoside (Inhaled tobramycin)	Modified biofilm architecture (*SpA* binds *Psl*)	[28]
		β-lactams	LPS-free PA co-culture evolved phenotypes	[112]
		Aminoglycoside (tobramycin)	SCVs selected for Dependent on Agr QS system	[157]
***S. maltophilia (+PA)***	Increased	Aminoglycoside (tobramycin)	‘Hiding’ in mixed biofilm	[18]
***P. aeruginosa* (+*SM)***	Increased	B-lactams (imipenem) Cephalosporin (ceftazidime)	SM secretion of β-lactamases, L1, and L2	[168]
	Increased	Polymyxins	SM DFS enhances PA resistance	[111]
***Prevotella (+PA)***	Increased	β-lactams (ceftazidime)	*P. melaninogenica* production of β-lactamases	[169]
***P. aeruginosa* (+*AF)***	Increased	NA	Increased the relative abundance of PA OMPs	[170]
	Increased	Cephalosporin (Cefepime)	‘Hiding’ in mixed biofilm	[21]
***P. aeruginosa (+CA)***	Increased	NA	CA enhances PA ECM production	[171]
	Increased	β-lactams (Meropenem)	CA ECM polysaccharides protect *PA*	[172]

PA = *P. aeruginosa*, SA = *S. aureus*, SM = *S. maltophilia*, AF = *A. fumigatus*, CA = *C. albicans*, SCV = small colony variant, HQNO = 4-hydroxy-2-heptylquinoline-N-oxide, HAQ = 4-hydroxy-2-, QS = quorum sensing, ATP = adenosine triphosphate, LPS = lipopolysaccharide, DFS = diffusible signal factor, OMP = outer membrane proteins, ECM = extracellular matrix, NA = not applicable.

## Data Availability

Data sharing not applicable. No new data were created or analysed in this study.
